# Toward Red Light Emitters Based on InGaN-Containing Short-Period Superlattices with InGaN Buffers

**DOI:** 10.3390/ma16237386

**Published:** 2023-11-27

**Authors:** Grzegorz Staszczak, Iza Gorczyca, Ewa Grzanka, Julita Smalc-Koziorowska, Grzegorz Targowski, Tadeusz Suski

**Affiliations:** 1 Institute of High Pressure Physics, Polish Academy of Sciences, Sokolowska 29/37, 01-142 Warsaw, Poland; 2 TopGaN Ltd., Sokolowska 29/37, 01-142 Warsaw, Poland

**Keywords:** red emission, superlattices, III-nitride semiconductors, quantum wells, band structure

## Abstract

In order to shift the light emission of nitride quantum structures towards the red color, the technological problem of low In incorporation in InGaN−based heterostructures has to be solved. To overcome this problem, we consider superlattices grown on InGaN buffers with different In content. Based on the comparison of the calculated ab initio superlattice band gaps with the photoluminescence emission energies obtained from the measurements on the specially designed samples grown by metal-organic vapor phase epitaxy, it is shown that by changing the superlattice parameters and the composition of the buffer structures, the light emission can be shifted to lower energies by about 167 nm (0.72 eV) in comparison to the case of a similar type of superlattices grown on GaN substrate. The importance of using superlattices to achieve red emission and the critical role of the InGaN buffer are demonstrated.

## 1. Introduction

III-V nitride semiconductors have a significant impact on a wide range of device applications. Efficient light generation enables the construction of a variety of visible and UV LEDs, micro-LEDs, and blue and green laser diodes. Nitride materials have also gained importance in electronics, enabling the promising development of high-power transistors, high electron mobility transistors, and vertical transistors. More detailed descriptions of some specific applications of nitride semiconductors can be found in Refs. [1,2,3,4,5].

Systems based on InGaN/GaN seem to be excellent material for the realization of such devices. InGaN/GaN quantum structures may be attractive for use in next-generation full-color micro-light-emitting diodes for red, green, and blue (RGB) displays, as they hypothetically cover the energy range from 3.5 eV to 0.7 eV [6,7]. On the other hand, there are some properties of InN-containing nitride alloys and quantum structures that also have a negative impact on the applications, creating problems that need to be solved. One of them is the effect of In clustering in InGaN and InAlN alloys, which leads to an anomalously large bowing of the band gap and its pressure coefficient. An explanation of the observed anomalous effects based on a density of states (DOS) and lattice relaxation analysis has been presented in Ref. [8]. The tendency of In atoms to form clusters leads to the large scattering of the experimentally measured emission energies; they can cover a significantly large energy range, indicating considerable differences in the In clustering characteristics for different samples. This effect often reflects the role of different growth conditions, such as growth pressure and temperature.

The next negative effect on applications consists of the presence of a built-in electric field in polar InGaN QWs with thicknesses above 3 nm. It leads to reduced light emission efficiency. However, in SLs with very thin QWs, the detrimental effect of the internal electric field is reduced.

Another “undesirable” property of InGaN alloys is their low stability. This is due to the significant differences in the lattice constants of GaN and InN (e.g., [9]). To obtain blue emission, ~18% of In is required, while for green emitters, introducing ~25% of In to QW is necessary. An even higher concentration of In is required for red light InGaN/GaN emitters. In practice, there are serious difficulties in realizing this idea. The large lattice mismatch between InN and GaN and the resulting phase separation effect make it difficult to grow high-quality In_x_Ga_1−x_N layers with high In content (x > 0.25). In_x_Ga_1−x_N quantum wells (QWs) also suffer from induced high strain due to lattice mismatch, and it is the source of deterioration of the structural quality of the grown InGaN/GaN heterostructures with high In-content. A high density of lattice mismatch-related defects is responsible for unwanted nonradiative electron–hole recombination, which significantly reduces the efficiency of epitaxially grown emitters with high In content in their active region.

To mitigate the lattice mismatch problem in InGaN alloys, the idea of InN/GaN superlattices (SLs) was introduced [10]. However, the problem of growing InGaN−based SLs with high In content in a QW arose. There is experimental evidence [11], confirmed by theory [12], that it is not possible to grow InGaN/GaN SL with higher In content (more than 33% of In).

The most promising methods of minimizing the above obstacle are based on proper strain engineering. Various efforts have been undertaken with the purpose of reducing the lattice mismatch between active InGaN regions and underlying layers. A comprehensive overview of the related attempts was presented by D. Iida and K. Ohkawa [13]. Most of them apply different underlayers to form the kind of pseudo-substrates, which are often treated as strain compensating buffers. In particular, Iida et al. [14] proposed the use of the thick GaN and AlGaN underlayers resulting in lowering in-plane stress. This approach makes it possible to obtain the efficient emission at 633 nm (1.96 eV). The idea described in refs. [15,16] is based on the use of compatible GaN on porous GaN pseudo-substrates for the growth of the emitter active elements. Another interesting solution was proposed by Dussaigne et al. [17]. Red electroluminescence was achieved using the full InGaN structure, with the InGaNOS substrate as a relaxed pseudosubstrate. Reduced strain was achieved by growing a red LED on the buried oxide.

In our paper, we propose another promising method of obtaining red emission. It consists of the use of nitride short-period superlattices (SLs) instead of the commonly used InGaN/GaN multi quantum wells (MQWs). Short−period SLs are layered quantum structures that consist of a small number of atomic monolayers (MLs) of quantum wells (QWs) and quantum barriers (QBs). The main advantages of using short−period SLs are (i) the reduction of “non-radiative” defects caused by lattice mismatch, (ii) the ability to precisely control the bandgap/emission wavelength, and (iii) the reduction of the built-in electric field and its screening by the emitter current [18], which leads to an unpredictable blue shift of the emission energy. 

Moreover, the use of SLs as the active region could help eliminate most of these problems that inhibit the development of red nitride emitters. In particular, it avoids the decomposition problems faced by “standard” thick InGaN/GaN QWs with high indium content [19,20]. 

The first short−period SL structures proposed by Yoshikawa et al. [10] were the *m*InN/*n*GaN SLs, where *m* and *n* denote the number of InN and GaN atomic layers, respectively. Theoretical predictions showed that the bandgap of such *m*InN/*n*GaN SLs could be varied over a wide range of energies/wavelengths by using different numbers for *m*, and *n*. Even the closing of the energy gap, E_g_, was predicted [21,22]. Unfortunately, detailed studies of photoluminescence and its pressure dependences have shown that the growing InN/GaN superlattices is extremely difficult from a technological point of view. It was found that instead of the intentionally grown superlattice of the single InN QW layer (as the light-emitting element) and multiple GaN layers (as the QB), the QW was actually formed by a single layer of In_0.33_Ga_0.67_N alloy [11].

Theoretical studies [12] confirmed that pseudomorphic growth of InN on GaN substrate is not possible and that there is a limit to the maximum In content in pseudomorphically grown InGaN on GaN due to lattice mismatch and high strain energy. On the other hand, the theory suggests that InN growth is possible on substrates with a higher “in-plane” lattice parameter than this corresponding to GaN. Siekacz et al. [23] studied InGaN/GaN short period SLs grown by MBE on partially relaxed InGaN buffers with increased lattice constant. They found that increasing the substrate a-lattice parameter from 3.183 to 3.216 Å resulted in an increase in the PL emission wavelength from 379 nm (3.27 eV) to 419 nm (2.96 eV) due to the increased In content of one ML thick InGaN QW. This was attributed to the reduced lattice mismatch between substrate and InGaN QW, allowing for more indium incorporation. This interpretation was confirmed by Even et al. [24] and Sharma et al. [25].

In this work, we have performed the detailed theoretical study of the bandgap behavior in *m*In_x_Ga_1−x_N/*n*In_y_Ga_1−y_N SLs with different numbers, *m*, *n*, of atomic layers and grown on different In_z_Ga_1−z_N substrates (called later buffers). General trends in the band gap behavior are discussed. The results indicate that in an effort to obtain red light emission, the recipe is to grow In_x_Ga_1−x_N/In_y_Ga_1−y_N SLs with In_z_Ga_1−z_N buffer consisting of the highest possible total In-content (x + y + z). 

To compare our theoretical results with the experimental data, a set of *m*In_x_Ga_1−x_N/*n*In_y_Ga_1−y_N SL samples was grown by the MOVPE (metal organic vapor phase epitaxy) method on In_z_Ga_1−z_N substrates. The obtained PL emission was in the range from about 441 nm (2.81 eV for *2*In_x_Ga_1−x_N/*n*In_y_Ga_1−y_N SLs grown on GaN substrate) to about 633 nm (1.96 eV for the same type of SLs grown on In_0.20_Ga_0.80_N substrate), in reasonably good agreement with the theoretical results, taking into account all the uncertainties in the characterization of the grown samples. We show that the use of In_x_Ga_1−x_N/In_y_Ga_1−y_N SLs grown on InGaN pseudosubstrates (i.e, buffers) allows to achieve the photoluminescence energy below 2 eV (about 630 nm).

## 2. Methods

### 2.1. Theoretical Calculations

To simulate the SL structure, supercells of up to 256 atoms were constructed. The positions of the atoms in the supercell were chosen to be as random as possible, and small changes in the atomic positions did not significantly affect the results. It has already been shown in our previous work that even SL calculations performed on smaller supercells (32, 64, or 128 atoms) lead to good agreement with the experimental results, for example, see the review paper [26].

**Figure 1:**
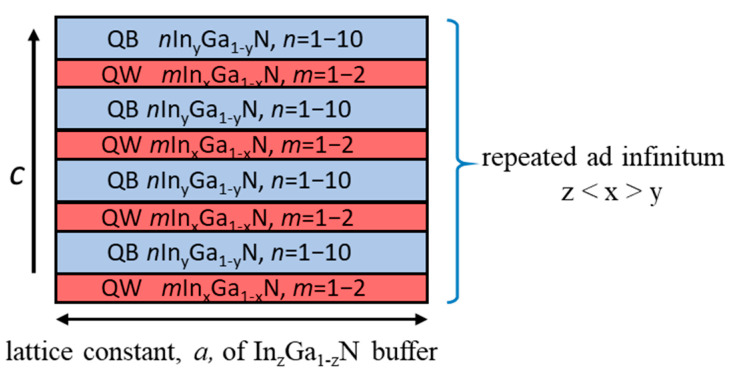
Scheme of the calculated *m*In_x_Ga_1−x_N/*n*In_y_Ga_1−y_N SLs grown on thick buffer layer. QW layers are in red, and the QB layers are in blue color.

A typical SL structure corresponding to the considered SLs is shown in Figure 1.

We assumed that the investigated SLs are grown pseudomorphically on a pseudosubstrate (i.e., buffer). Consequently, we kept the lattice constant perpendicular to the growth direction equal to the buffer lattice constant. The lattice constant parallel to the growth direction and the relaxed positions of the atoms in the supercell were obtained by ab initio calculations of the SLs band structure. 

The computations were carried out in two steps, in which two different calculation schemes were applied. In the first step, the atomic coordinates in the supercell were determined by minimizing the Hellmann–Feynmann forces. For this task we used pseudopotentials as implemented in the Vienna Simulation Package (VASP) [27] with the Perdew–Zunger parameterization [28] of the Ceperley–Alder exchange correlation [29]. The converged results were obtained with an energy cutoff of 30 Ry, and the k-space integrations were performed by summing over a 5 × 5 × 5 mesh of Monkhorst–Pack special points.

Subsequently, in the second calculation step, the obtained relaxed atomic positions were used as input to the energy band structure calculations by the full potential (FP) version [30] of the linear muffin tin orbital (LMTO) method [31] with the band gap correction procedure (LDA + C) [32,33], as implemented in the FP LMTO code. In this method, the unit cell in a wurtzite structure contains four “real” atoms and four so-called “empty spheres”. The calculations were optimized by the choice of equal atomic-sphere radii for real atoms and different values for two types of empties. The “semicore” Ga 3d and In 4d states are treated as fully relaxed band states. 

The semi-empirical procedure (LDA + C) was used to correct the band gap values underestimated by LDA. It is a relatively simple correction scheme, but a bit more advanced than a rigid shift of the unoccupied bands (“scissor operator” correction procedure). It corrects not only the fundamental gap, but also the dispersion of the lowest conduction band CB and the gap values at other points in the Brillouin zone. In this procedure, additional external potentials, *V*(*r*), are introduced at the sites of the atoms [32]:

(1) $$V\left(r\right)=V_{0}\left(\frac{r_{0}}{r}\right)\exp\left[-{\left(\frac{r}{r_{0}}\right)}^{2}\right]$$

where *V*_0_ and *r*_0_ are adjustable parameters. The potentials are sharply peaked at the nuclear positions and produce “artificial Darwin shifts”, i.e., they push s-states upward in energy. This method of correcting Eg errors caused by the LDA was originally developed and extensively used in LMTO calculations [32,33,34,35,36,37], then in a linear augmented plane wave (LAPW) framework, and also, subsequently in a pseudopotential method [38].

The parameters used in the external potentials are transferable. They are specific to the atomic species, so they can be determined for binary compounds and then applied to other systems such as alloys and heterostructures. They also remain unchanged as volume and composition are varied [32,33,34,35,36]. They are determined by fitting to the experimental band gaps and to the results of spectroscopic ellipsometry, for both, binaries and alloys.

Optimal values of the LDA + C parameters obtained from the adjustment procedure are the following: *V_0_*(In) = *V_0_*(N) = 0, *V_0_*(Ga) = 900 Ry, *V_0_*(Al) = 990 Ry, with the range parameter r_0_ = 0.015 a.u. for all atoms. For the empty spheres, *V_e_* = 0.60 Ry, being r-independent. Subsequently, we used the above parameters in the LMTO band structure calculations of nitride alloys and SLs.

Further details of the LDA-LMTO calculations are given elsewhere [26,33].

### 2.2. Structural Investigation of the Samples

Thickness, In content, and structural quality of the prepared structures, were checked by X-ray Diffraction (XRD) on Empyrean Panalytical, and for selected samples also by Transmission Electron Microscopy (TEM) on FEI Titan 80–300 microscope, with spectral resolution 1.4 Å in scanning (STEM) operation mode.

### 2.3. Optical Measurements

Continuous wave photoluminescence (cw-PL) experiments were performed by using a DPSS (diode-pumped solid-state laser) laser emitting at 320 nm with a power density of approximately 53 kW/cm^2^. The emission spectra of the investigated samples was collected by a T6400 spectrometer (Horiba Jobin Yvon, Warsaw, Poland) and detected by a charge-coupled device camera (CCD) in a backscattering configuration. The samples were attached to the cold-head of an optical Helium closed-cycle cryostat, which allowed for the temperature control ranging from 20 K to 300 K. The measurements were carried out at a temperature of 20 K.

## 3. Results and Discussion

### 3.1. Theoretical Results

InGaN-based short period SLs of the *m*In_x_Ga_1−x_N/*n*In_y_Ga_1−y_N (x > y) type, where *m* is the number of QW MLs and *n* is the number of QB MLs, were studied by ab initio calculations. We assumed that they are grown pseudomorphically on different In_z_Ga_1−z_N buffer layers, adopting their lattice constant perpendicular to the growth direction.

For the more detailed analysis, we selected SLs with mIn_0.33_Ga_0.67_N QW, since they contain the highest In concentration available for this type of heterostructures [11]. The selected SLs have all possible combinations of the following QBs and buffers: GaN, In_0.165_Ga_0.835_N, and In_0.25_Ga_0.75_N.

**Figure 2:**
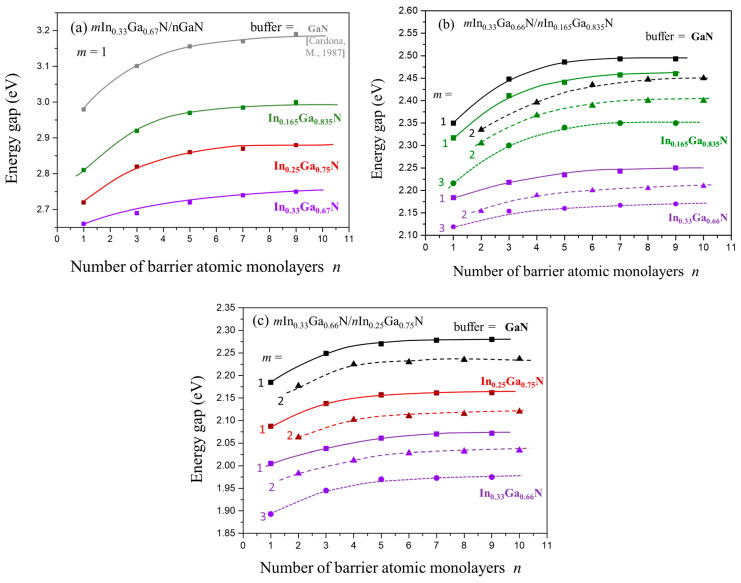
(Calculated band gaps, E_g_, vs. number of barrier MLs, *n*, for a set of SLs: (**a**) *m*In_0.33_Ga_0.67_N/*n*GaN, (**b**) *m*In_0.33_Ga_0.67_N/*n*In_0.165_Ga_0.835_N, (**c**) *m*In_0.33_Ga_0.67_N/*n*In_0.25_Ga_0.75_N. Each group of curves, marked with a different color, corresponds to a particular InGaN buffer (named on the right side of each figure).

From the electronic band structure calculations, we obtained the energy band gap values for the considered SLs. Figure 2a–c show the obtained band gap values versus the number of QB MLs, n, and for the number of QW MLs, m, up to 3. For mIn_0.33_Ga_0.67_N/GaN SLs, only the results for m = 1 are presented. 

Considering the dependence of the band gap values on the QB thicknesses, we observe that for a given SL with the number of QW MLs, m, E_g_ increases up to the number of QB MLs, n = 5, and then saturates. An increase in E_g_ with the number of layers in the barrier varies from 0.05 to 0.1 eV depending on the buffer—for buffers with higher In content, this difference is smaller (the curves illustrating the dependence on the number of QB MLs are flatter). 

By analyzing Figure 2, we can estimate the sensitivity of the band gap values to the thickness of QWs and QB and to the In content in QBs and buffers. We observe the following:

- **Reduction of E_g_ with increasing number of QW layers**. The observed gap reduction between SLs with 1 and 3 QW monolayers is in the order of 0.1 eV. Apart from the reduced hybridization effect, for thicker QWs, the built-in electric field starts to play a more significant role, leading to a decrease in the band gap values. The latter effect is described by Quantum Confined Stark Effect.
- **Reduction of E_g_ with increasing In content in the QB**. The obtained gap reduction (for SLs with the same QW and buffer) is between 0.7 and 0.9 eV, depending on the case. We can explain this effect by the hybridization of the QW and QB wave functions, which is dominant in SLs with thin QWs.

**Figure 3:**
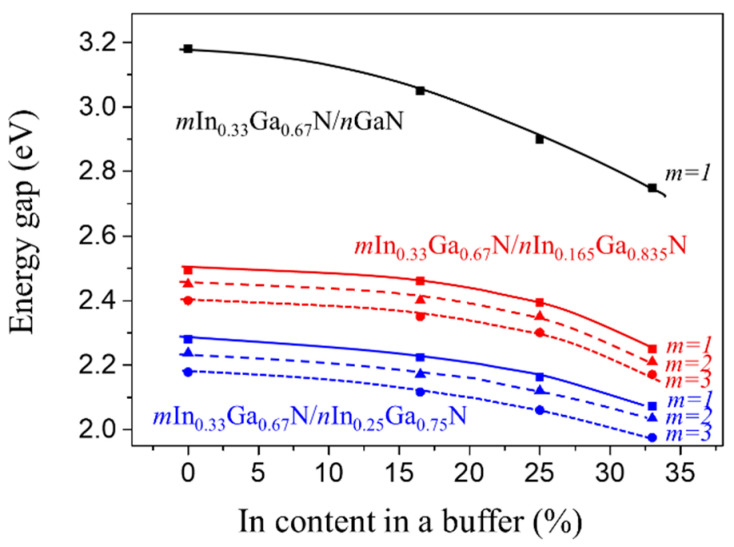
Calculated band gap for *m*In_0.33_Ga_0.67_N/*n*GaN, mIn_0.33_Ga_0.67_N/nIn_0.165_Ga_0.835_N, and mIn_0.33_Ga_0.67_N/nIn_0.25_Ga_0.75_N SLs vs. In content in a buffer. Different colours denotes indium content in the buffer layer.

The above two points have been illustrated and discussed in the previous papers [39,40]. In the present paper, we can concentrate on the following:

- **Reduction of E_g_ with increasing In content in the buffer.** The reduction of the band gap values between the same SLs with GaN and In_0.33_Ga_0.67_N buffers results in a gap reduction of about 0.4 eV for In_0.33_Ga_0.67_N/GaN, and about 0.2 eV for the other two SLs (Figure 2b,c). For a clearer illustration, we present in Figure 3 the dependence of the band gaps on the content of In in a buffer for three considered SLs. We observe a strong nonlinear decrease in the band gap values with increasing In content in a buffer, the higher the In content in the buffer, the more pronounced the band gap decrease.

The reduction of E_g_ with increasing In content in the buffers is mainly caused by a smaller misfit strain present in the QW connected with a higher amount of In in the InGaN buffer layer.

Further into the paper, we will confront the above findings with the experimental results obtained from the measurements performed on the selected structures grown by the MOVPE method.

### 3.2. Experimental Results

#### 3.2.1. Structural Characterization of the Samples

To confirm the results of our calculations, a series of InGaN/InGaN SLs with different geometries was prepared. All samples were grown by the MOVPE method. The layers were deposited on sapphire with 70 nm thick InGaN buffer layers with about 17% and 20% indium content (hereafter referred to as type A and B, respectively). Trimethylindium (TMIn), ammonia (NH_3_), and triethylgallium (TEGa) were used as indium and gallium precursors, respectively. Nitrogen (N_2_) was used as the carrier gas. The sample growth temperature was controlled by a thermocouple placed under the graphite susceptor. The active region of the sample was grown under constant pressure and temperature (450 mbar and 780 °C, respectively).

**Figure 4:**
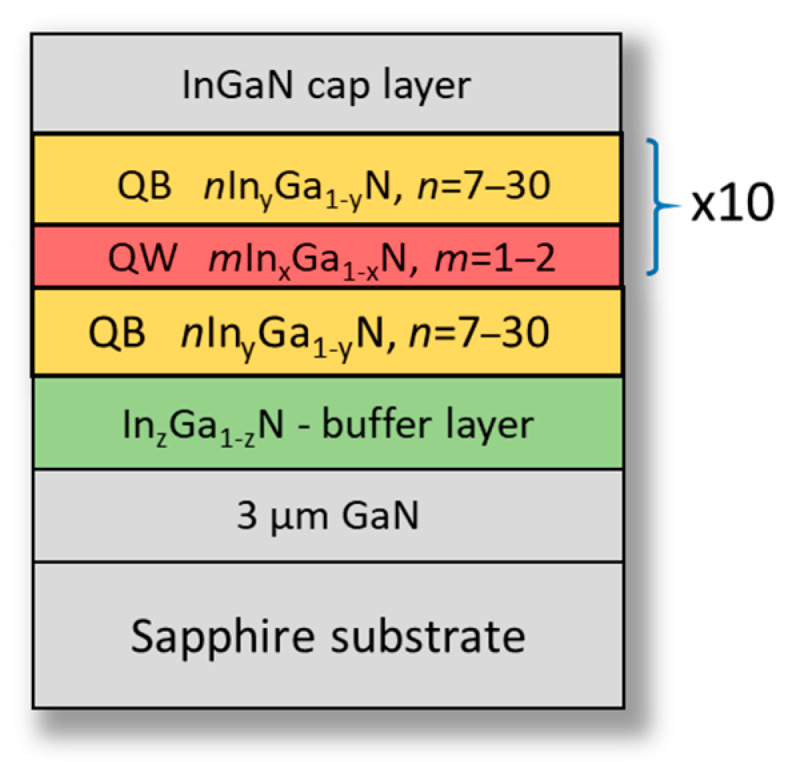
Scheme of the investigated *m*In_x_Ga_1−x_N/*n*In_y_Ga_1−y_N superlattices grown on buffer layer with z~0.17, and z~0.2 of In content; *m, n* denotes the number of QW and QB MLs, respectively. The thickness of one ML is around 0.26 nm.

The sequence of QWs and QBs was repeated 10 times, and the average indium content in the QW was about 28% for type A, while in the case of samples B, the indium content was about 31%. The observed higher indium content for sample B is related to the use of buffer with higher In content (lower mismatch between QWs and QBs). The InGaN buffer layer was grown at 780 °C. The selected structures on InGaN buffers studied in this work have been compared with their counterparts grown on GaN substrate without buffer layer and described elsewhere [39,40]. The scheme of samples structure is shown on Figure 4.

**Figure 5:**
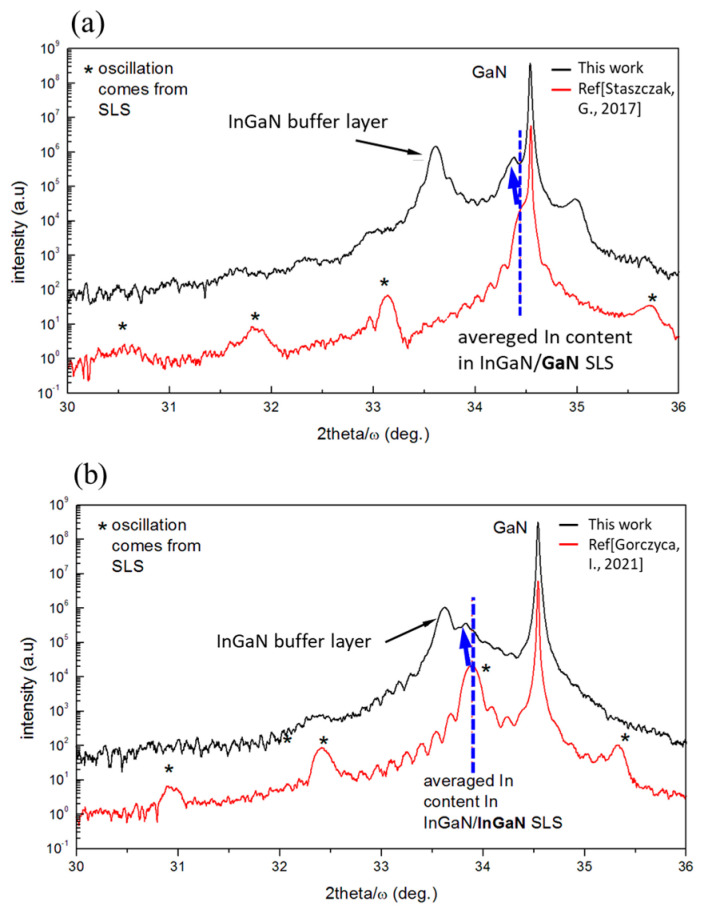
The XRD patterns depict the characteristics of two samples: (**a**) sample A1 of InGaN/GaN SL (no indium in QBs), and (**b**) sample A3 of InGaN/InGaN SL (with 15% In in QBs). The black line corresponds to the sample grown on an InGaN buffer, while the red line represents the sample grown on GaN (see Refs. [39,40] respectively). The blue arrow indicates the change in the average In content in the SLs. The dotted blue line shows the 0−order oscillation position corresponding to average In-content in SLs.

**Figure 6:**
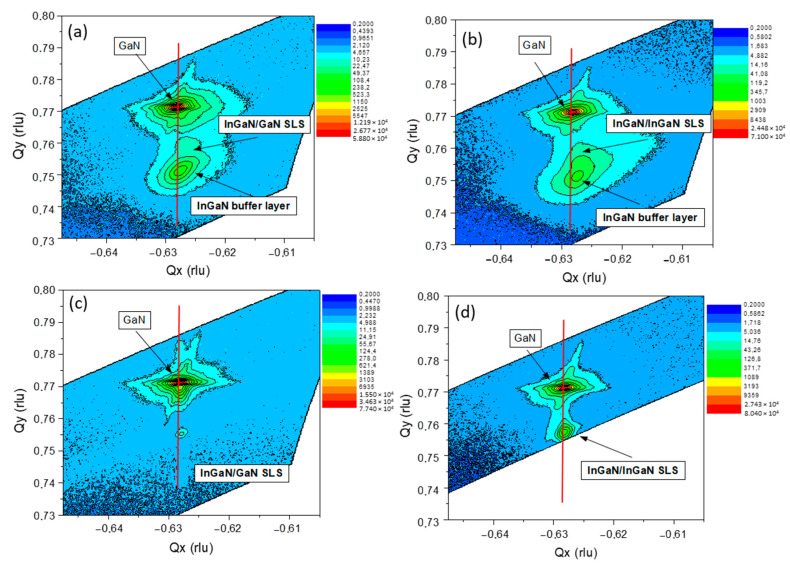
Comparison of XRD reciprocal space maps around the asymmetric GaN (10–14) reflection for (**a**) sample A1 and (**b**) sample A3 with the corresponding structures grown on GaN (**c**,**d**).

**Figure 7:**
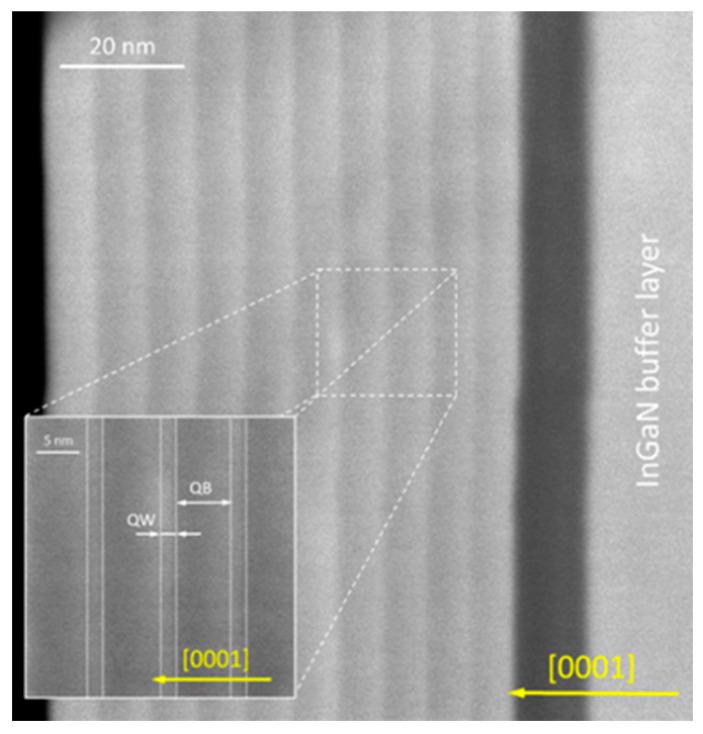
Cross-sectional TEM image of the *m*InGaN/*n*InGaN SLs with *m* = 2 and *n* = 30 MLs grown on In_0.17_Ga_0.72_N buffer layer (sample A3).

Basic structural parameters such as In content, layer thickness, strain relaxation, and structural quality of the selected structures were checked by high-resolution X-ray diffraction (HR XRD) (Empyrean Panalytical, UK) and also by TEM (Titan 80-300, with spectral resolution 1.4 Å FEI, Holland,) in scanning mode (STEM), showing good agreement with the intentionally chosen parameters. XRD 2 theta/omega patterns, reciprocal space maps, and TEM cross sections for selected samples are shown below (Figure 5, Figure 6 and Figure 7). The structural parameters of the investigated SL structures are summarized in Table 1. All considered samples are divided into two types, marked by A and B, corresponding to different buffer materials. Intentionally, all samples should consist of up to 2 MLs in QWs and different thicknesses of QBs. HR XRD measurements were performed on all samples listed in Table 1 to obtain 2theta/ω patterns. The obtained results allowed us to determine the indium content and the widths of the QWs and QBs. Figure 5 shows a comparison between the exemplary patterns of two selected samples: (a) sample A1 and (b) sample A3 (black lines) with the corresponding samples grown on GaN (red lines) [39,40].

The introduction of the InGaN buffer led to an increase in the indium content compared to their counterparts grown on GaN. The average indium content increased by about 6–7% in the QWs and 2–3% in the QBs. However, the use of the InGaN buffer layer significantly reduces the structural quality of the superlattice, which can be observed by the reduction of the intensity of the peaks (indicated by stars) originating from the SL structure in the XRD patterns (Figure 5). To verify the assumption that the investigated SL structures are grown pseudomorphically on InGaN substrate, we performed the measurement of the reciprocal space map (RSM) of In_x_Ga_1−x_N/GaN (sample A1) and In_x_Ga_1−x_N/In_y_Ga_1−y_N (sample A3) around the (10–14) reflection (Figure 6a and Figure 6b, respectively). For both types of structures, the SLs are strained to the InGaN buffer. For comparison, in analogous GaN−buffer grown structures, the SLs are fully strained to the GaN layer (Figure 6c,d).

In TEM studies, we observed that the interface between the lower InGaN QB and the InGaN QW is sharply defined, while the interface between the upper InGaN QW and the InGaN QB is rough due to variations in QW thickness and In composition (Figure 7). This phenomenon has been previously observed in InGaN/GaN and InGaN/InGaN SLs grown on GaN substrates [39,40]. The low contrast between the InGaN QB and the InGaN QW and the uneven surface of the TEM sample make the SL structure less visible in the STEM images. However, the periodicity becomes more apparent in the thicker areas of the sample where the STEM signal comes from a larger sample volume and provides a more averaged image of the structure.

#### 3.2.2. Optical Results

Identification of the peaks was made as follows: comparing the PL spectra of all samples, the peaks at ~2.8 eV (for all samples A with 17% In in a buffer) and ~2.7 eV (for all samples B with 20% In in a buffer) can be identified as coming from the buffer. The second peak moves down in energy from 2.7 eV (sample A2) to about 1.9 eV (sample B2) with increasing In content in the SL. We can observe that the intensity of the emission from the SLs is much higher than that from the buffer.

**Figure 8:**
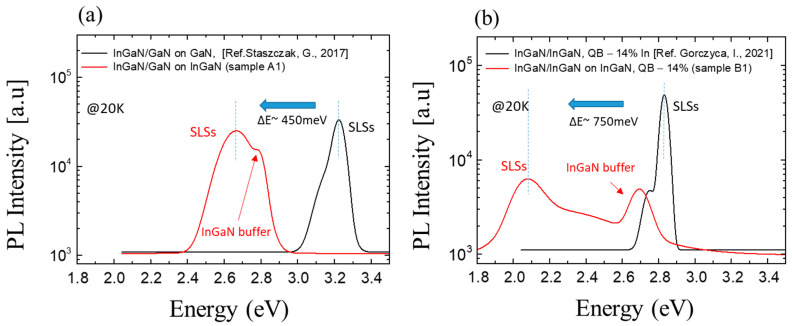
Comparison of PL spectra @20K of the selected In_x_Ga_1−x_N/In_y_Ga_1−y_N SLs: (**a**) sample A1 without indium in QBs (y = 0) and (**b**) sample B1 with indium in QBs (y = 0.17). Both structures were grown on GaN layers (black line) and InGaN buffer layers (red line).

It can be observed that the introduction of an InGaN buffer layer significantly reduces the emission energy for both SL structures. The reduction at T = 20 K is about 0.45 eV for type A structures and about 0.75 eV for type B structures (Figure 8a and Figure 8b, respectively). The experimentally observed reduction in E_PL_ is most likely related to a decrease in internal strain due to a lower mismatch between QWs-QBs and the substrate. This leads to a decrease in the built-in electric field. The spectra were smoothed due to Fabry–Perot oscillations. In the case of structure type B, the observed decrease in luminescence intensity is caused by a significant decrease in structural quality. Relaxation of the buffer layer generates a significant amount of non-radiative recombination centers that influence the emission intensity. 

### 3.3. Comparison between Theory and Experiment

**Figure 9:**
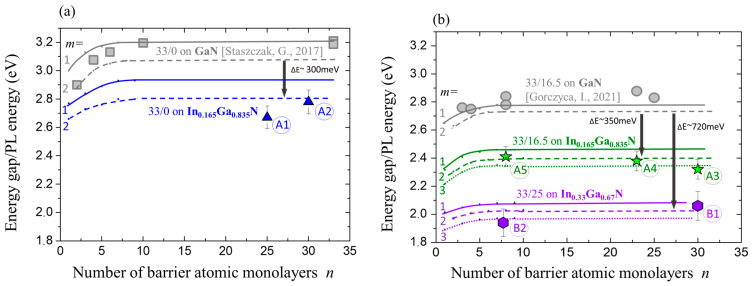
Comparison of the calculated SL band gaps, E_g_, and PL emission energies. For SLs, instead of *m*In_x_Ga_1−x_N/*n*In_y_Ga_1−y_N we used the short notation: x/y. (**a**) SLs 33/0 on buffers: GaN and In_0.165_Ga_0.835_N (blue line). (**b**) SLs 33/16.5 (green line) and 33/25 (violet line) on buffers: GaN and In_0.165_Ga_0.835_N and In_0.33_Ga_0.67_N. Results of our previous calculations on GaN buffers are marked in grey. Triangles, stars, and hexagons represent experimental results for the investigated samples.

In Figure 9a, a comparison of the calculated SL band gaps, E_g_, and PL emission energies, E_PL_, for three selected SLs (marked in blue, green, and red) is shown. For SLs, we used the short notation: x/y instead of *m*In_x_Ga_1−x_N/*n*In_y_Ga_1−y_N. The results of our previous calculations and measurements performed on SLs grown on GaN buffers [39,40] are also shown and they are marked in grey.

Looking at Figure 9 and taking into account differences between SLs with m = 2 as corresponding to the character of measured SL samples, we can observe the following:

(i) Comparing SLs 33/0 (Figure 9a) grown on GaN and on an InGaN buffer, we observe a downshift of the E_g_ energy of about 0.3 eV and between 0.3 and 0.4 eV (E_PL_).
(ii) Comparing SLs 33/16.5 grown on GaN and on an In_0.165_Ga_0.835_N buffer, we observe a downshift of the E_g_ and E_PL_ of about 0.35 eV (Figure 8b).
(iii) Comparing SLs 33/16.5 grown on GaN and 33/25 grown on an In_0.33_Ga_0.67_N buffer, we observe a downshift of the E_g_ of about 0.72 eV and a downshift of the E_PL_ between 0.72 and 0.75 eV (Figure 9b).
(iv) The lowest band gap energy obtained for 33/25 grown on In_0.33_Ga_0.33_N SL is slightly below 2 eV, in agreement with the experimental results.
(v) It is suggestive to formulate the general observation that can be derived from our studies: that the total amount of In in an SL, including a buffer, determines the magnitude of E_g_/E_PL_.

The calculations and experimental results are in reasonably good agreement. Nevertheless, an increase in the average total In content within the structure (QWs, QBs, buffers) leads to a larger scatter of experimental data points compared to the theoretical data. This phenomenon is mainly due to the variations in width and In content in the QWs and/or QBs depending on the growth conditions.

## 4. Conclusions

In an attempt to solve the problem of low In incorporation in InGaN-based heterostructures, we have considered, both theoretically and experimentally, superlattices of the *m*In_x_Ga_1−x_N/*n*In_y_Ga_1−y_N (x > y) type grown on different In_z_Ga_1−z_N buffers. The specially designed samples were grown by MOVPE using buffers with 17% and 20% of In content. We have shown the following:

(a) Not only the amount of In in the QW and in QB, but also the amount of In in the buffer determines the magnitude of E_g_/E_PL_. The higher the In content of a buffer, the more pronounced the observed reduction in the energy gap values.
(b) Based on the comparison of the calculated ab initio band gaps of the SLs with the PL, the emission energies obtained from the measurements on the specially designed samples, it has been shown that by changing the In content in the buffer structures, the light emission can be shifted to lower energies by about 0.72 eV (167 nm) compared to the case of similar type of SLs grown on GaN substrate.

The experimental results presented in this paper are at an early stage. However, we believe that by optimizing the growth conditions of the buffer layers, and by using SLs with specially designed geometry, it is possible to obtain an emission that is even more shifted towards the red region of the spectrum. The calculations presented in this paper have been confirmed by experimental results, which show sufficiently good agreement with the calculations.

## Figures and Tables

**Table 1:** Characteristics of the investigated SLs. Sample name, In content in QW and QB, QW and QB thickness. The last column contains PL emission energies at 20K.

	Type of the Sample	XRD QW in Content ± 2 (%)	XRD QB in Content ± 2 (%)	QW Thickness (MLs) ± 0.5 ML	QB Thickness (MLs) ± 0.5 ML	E_PL_ (eV) at 20 (K)
InGaN buffer 17%	A1	28	0	2	25	2.67
	A2	28	0	2	30	2.78
	A3	28	15	2	30	2.37
	A4	28	15	2	23	2.38
	A5	28	15	2	8	2.41
InGaN buffer 20%	B1	31	17	2	30	2.07
	B2	31	18	2	8	1.94

## Data Availability

The datasets generated and analyzed during the current study are available from the corresponding author on reasonable request.
